# Personal stigma and use of mental health services among people with depression in a general population in Finland

**DOI:** 10.1186/1471-244X-11-52

**Published:** 2011-03-31

**Authors:** Esa Aromaa, Asko Tolvanen, Jyrki Tuulari, Kristian Wahlbeck

**Affiliations:** 1 Vaasa Hospital District and National Institute for Health and Welfare, Psychiatric Unit of Vaasa Central Hospital, Sarjakatu 2, Vaasa, FI- 65320, Finland; 2 Department of Psychology, University of Jyväskylä, P.O. Box 35, FI-40014, Finland; 3 South-Ostrobothnia Hospital District, Psychiatric Clinic of Lapua, Sairaalantie 9, FI-62100 Lapua, Finland; 4 National Institute for Health and Welfare, Psychiatric Unit of Vaasa Central Hospital, Sarjakatu 2, Vaasa, FI- 65320, Finland

## Abstract

**Background:**

A minority of people suffering from depression seek professional help for themselves. Stigmatizing attitudes are assumed to be one of the major barriers to help seeking but there is only limited evidence of this in large general population data sets. The aim of this study was to analyze the associations between mental health attitude statements and depression and their links to actual use of mental health services among those with depression.

**Methods:**

We used a large cross-sectional data set from a Finnish population survey (N = 5160). Attitudes were measured by scales which measured the belief that people with depression are responsible for their illness and their recovery and attitudes towards antidepressants. Desire for social distance was measured by a scale and depression with the Composite International Diagnostic Interview Short Form (CIDI-SF) instrument. Use of mental health services was measured by self-report.

**Results:**

On the social discrimination scale, people with depression showed more social tolerance towards people with mental problems. They also carried more positive views about antidepressants. Among those with depression, users of mental health services, as compared to non-users, carried less desire for social distance to people with mental health problems and more positive views about the effects of antidepressants. More severe depression predicted more active use of services.

**Conclusions:**

Although stronger discriminative intentions can reduce the use of mental health services, this does not necessarily prevent professional service use if depression is serious and views about antidepressant medication are realistic.

## Background

Unfortunately, only a minority of those who would benefit from professional treatment for depression actually seek it and many discontinue treatment prematurely. Only 34% of people with major depression in Finland seek professional help [1]. Similar results from other countries in Europe and the United States reveal the problem to be global [2,3].

Descriptive models, which try to explain service use in terms of the combined effects of socio-demographics (age, gender, education), access (income, insurance, availability of services) and severity of illness, have only modest power to predict the help-seeking of people with mental conditions [4]. Theoretical models on help-seeking behavior suggest that individual progress through several stages before seeking mental health treatment. They experience symptoms, try to evaluate their significance, assess if they can manage them by themselves or if treatment is required, assess the feasibility of and options for treatment, and decide whether to seek treatment [5]. Health belief theorists have shown that a rational consideration of the costs and benefits of participating in specific treatments may be an important factor when an individual decides to use services [6]. One such perceived cost to engaging in mental health services may be the risk of stigma. It has been suggested that many people hesitate to use mental health services because they do not want to be labeled a "mental patient" and want to avoid the negative consequences connected with stigma [7]. Among people with serious mental illnesses as well as nonpsychotic mental disorders, who perceived a need for help, the most commonly reported reasons for not seeking treatment were a will to solve the problem on their own and a hope that the problem would get better by itself [8,9].

There is conflicting empirical data about the effects of stigmatizing beliefs on seeking help from professionals for depression. Some studies have found a connection [10-13], while others have not [14-16].

One explanation for this could be the complexity of the concept of stigma and thus differences in measuring it. It has been demonstrated that some dimensions of stigma connected with mental illness were associated with potential care-seeking while others were not [13,17,18]. Another explanation for the mixed results may be different samples. Some studies use only people with depression in their samples while others take their samples from the general population.

Stigma related to mental health problems can be divided into perceived public stigma/stereotype awareness (participants' beliefs that in general people with mental illness are stigmatized in society), personal stigma/stereotype agreement (participants' personal beliefs about mental illness) and self-stigma (participants' view of their own mental illness)[19-21]. In particular, perceived stigma and self-stigma have relevance in the context of help-seeking. In many cases, they seem to interact [7,22]. Some authors differentiate a perceived public stigma associated with seeking professional services from the perceived public stigma associated with mental illness [22] and have developed scales to measure specifically this stigma component.

An issue closely related to attitudes towards people with psychiatric conditions, mental health professionals and the service system, is people's knowledge about mental disorders, remedies and services. In a review about public beliefs regarding treatment of depression as well as on other psychiatric conditions, psychosocial interventions were predominantly perceived as favorable, while negative views prevailed about pharmacological treatments [23]. In general, without psychiatric treatment, the course of schizophrenia is seen more pessimistically than in the case of depression. Conversely, as long as appropriate treatment is provided, the prognosis for both disorders is assessed as quite optimistic [23]. Given that evidence exists of possibilities to improve people's awareness and knowledge about depression, public beliefs may over time move closer to those of health professionals [24]. Nevertheless, it is still an open question if this would lead to an increase in actual help-seeking on a population level.

So far only a few studies have explored the connection between depression-related attitudes and actual help-seeking. Usually respondents have been asked about their intentions to seek professional help. Another methodological limitation has been the use of small student samples, with large population samples lacking.

In this paper our first aim was to look at whether people with depressive symptoms in a general population carry different kinds of stigmatizing attitudes compared with non-depressive respondents. Our second aim was to study if there is any connection between attitudes and the actual use of mental health services among those with depression.

## Methods

The survey questionnaire was mailed to 10000 persons aged 15-80 who were randomly selected from the Finnish Population Register and resided in four hospital catchment areas in western Finland. The overall response rate was 51.6% without any incentives or reminders. Overall, the response rate among females was 60% and among males 43%, with the highest response rate in the 50-70 age group. The average age of the respondents was 50.6 (SD 17.3) years. Overall, 16.5% of the respondents were Swedish-speakers. The lowest response rate was among Finnish-speaking men (42.1%) and the highest among Swedish-speaking women (68.8%). Population means and percentages were weighted according to age, gender, language and hospital area to ensure representativeness of the general population in the research regions. According to Finnish legislation (Medical Research Act 488/1999, (English translation available at http://www.finlex.fi/en/laki/kaannokset/1999/en19990488), ethical approval is needed only for medical research, that is defined as research involving interventions. Thus ethical approval is not needed for e.g. register-based research, opinion polls or anonymous general population postal surveys. The current study was part of a repeated anonymous general population postal survey, performed every three years. Neither this study, nor the repeated general population survey, are "medical research" according to Finnish legislation, and statutory ethical committees will not deal with studies that are perceived as not being "medical research". Thus ethical approval was not needed, nor applied for.

The postal survey questionnaire was 8 pages long with 36 questions, many of which included several parts, giving over 140 variables in total http://info.stakes.fi/vaasanosaamiskeskus/EN/researchanddevelopment/researchanddevelopment.htm. In this paper we applied the following variables:

The socio-demographic background variables were gender (coded as 1 = male, 2 = female) and age (year of birth).

Respondents who fulfilled self-reported criteria for major depressive disorder (MDD) according to the Diagnostic and Statistical Manual, fourth edition (DSM-IV) within the last twelve months were identified using questions from the Composite International Diagnostic Interview Short Form (CIDI-SF)[25]. With this instrument we can both estimate the occurence of depression and its severity.

Professional help-seeking was ascertained by asking: "Have you during the past 12 months used any health services because of mental problems?". Response choices included "yes" and "no" (coded as 1 = used services, 2 = not used services). We also asked about the use of different types of mental health services by asking: "During the last 12 months, did you seek help from any of the following service institutions in respect of a mental health problem" and gave respondents 12 alternatives.

Sixteen statements exploring attitudes to and stereotypes of mental health were developed based on earlier studies measuring public attitudes towards mental health problems and also on researchers' clinical experience (Table 1). Eight of the statements related to mental health problems in general and eight to depression only. Three of the statements referred to perceived public stigma/stereotype awareness and the rest to personal stigma/stereotype agreement. A four-point rating scale was used with the response alternatives: "strongly disagree", "disagree", "agree" and "strongly agree"

**Table 1:** Results of the Principal Components Analysis (followed by Varimax rotation) applied to the 16 items data collected in 5160 population sample

Items	I	II	III	IV
People with depression have caused their problems themselves.^2^	0.68			
Depression is a sign of failure.^2^	0.68			
Depressed people should pull themselves together.^2^	0.67			
Mental health problems are a sign of weakness and sensitivity.^2^	0.61			
Depression is not a real disorder.^2^	0.59			
Patients suffering from mental illnesses are unpredictable.^2^	0.31			
If one tells about his/her mental problems, all friends will leave him/her.^1^		0.67		
If the employer finds out that the employee is suffering from mental illness, the employment will be in jeopardy.^1^		0.64		
The professionals in health care do not take mental problems seriously.^1^		0.59		
Depression can be considered as a shameful and stigmatizing disease.^2^		0.57		
It is difficult to talk with a person who suffers from mental illness.		0.45		0.31
Antidepressants are not addictive.^2^			-0.78	
Antidepressants have plenty of side effects.^2^			0.68	
Society should invest more in community care instead of hospital care.^2^				-0.81
Depression can't be treated.^2^	0.37			0.39
You don't recover from mental problems.^2^	0.32	0.34		0.39

^1 ^Statements refer to perceived public stigma/stereotype awareness.

^2 ^Statements refer to personal stigma/stereotype agreement

Our first scale in this analysis, "Depression is a matter of will", measures negative stereotypes about people with depression and the belief that people with depression are responsible for their illness and their recovery. It was built from following five statements measuring personal stigma:

1. "Depression is a sign of failure"

2. "People with depression have caused their problems themselves"

3. "Depressed people should pull themselves together"

4. "Mental health problems are a sign of weakness and sensitivity"

5. "Depression is not a real disorder"

These statements were extracted by principal component analysis (PCA)[26]. Prior to performing the PCA the suitability of the data for factor analysis was assessed. Inspection of the correlation matrix revealed the presence of many coefficients of 0.3 and above. The Kaiser-Meyer-Olkin value was 0.830, above the minimum recommended value of 0.6 and the Bartlett's Test of Sphericity reached statistical significance (p = 0.000), suggesting that a factor analysis was appropriate.

The PCA revealed the presence of four components with eigenvalues exceeding 1, explaining 21.7%, 9.3%, 8.1% and 6.6% of the variance respectively (Table 1). This model accounted for 45.7% of the total variance. To aid in the interpretation of these four components, a Varimax rotation was performed. An identical PCA was performed three years earlier in a similar population survey and it identified exactly the same structure of four components. This analysis is reported elsewhere [27].

The main component, here called "*Depression is a matter of will"*, consisted of eight items and accounted for 21.7% of the variance. If the three items with low loadings ("Patients suffering from mental illness are unpredictable","Depression can't be treated" and "You don't recover from mental health problems") are excluded, we have a feasible five-item-scale with an internal consistency of 0.70 and inter-item correlations from 0.38 - 0.50. A high score on this scale indicates a belief that a person is responsible for the cause and course of his or her depression, and also capable of recovering from the illness if sufficiently strong-willed.

Our second attitude scale in this analysis, here called "*Antidepressant attitudes*" consisted of the two items in PCA component 3 and accounted for 8.1% of the variance. This 2-items scale has a very low internal consistency of 0.42 but because these items are highly correlated, we use them as a measure of antidepressant attitudes/knowledge in this analysis. A higher score on this scale indicates a belief that antidepressants are addictive and have plenty of side effects.

Our third attitude scale in this analysis, here called "Desire for Social Distance", reflects personal desire for social distance. This scale was constructed from a different set of items contained in the survey questionnaire and is based on respondents' expressed willingness in four different imaginary situations to be in contact with a person who has mental problems:

1. "Would you be willing to marry or be in a common law marriage with someone, who has mental problems?"

2. "Would you be willing to give your child into the care of someone who has mental problems?"

3. "Would you be willing to choose someone who has mental problems as your work colleague?"

4. "You find out that a rehabilitation centre for patients with mental illnesses is being planned in your neighborhood. Would you object to the plans?"

The fifth question "A person you know is committed to psychiatric hospital care. Would you be willing to visit him there?" was not included in the scale to make internal consistency stronger and because of its poor ability to differentiate. A higher total score means less willingness to be in contact with a person who has mental problems. The internal consistency of this scale was 0.70 (Cronbach's alpha).

The connections between depression (as measured by the CIDI-SF) and components of personal stigma (as measured by the "Depression is a matter of will"-scale, the "Desire for Social Distance"-scale and the "Antidepressant Attitudes" - scale) were analyzed using logistic regressions. Age and gender were entered in this model simultaneously with attitude components.

The relative effects of these three attitude scales on 12-month help- seeking among persons with depression were also analyzed using logistic regressions. Age and gender as well as the degree of depression were entered in this model simultaneously with attitude components. All analyses were carried out with SPSS 16 software.

## Results

The CIDI-SF identified 558 (10.9%) cases of major depression, using a twelve month prevalence definition. Of those 381 (68%) were women and 173 (32%) men. 221 (39.6%) of them had used health services during the last 12 months because of mental health problems. 55 (31.8%) men and 165 (43.3%) women had used mental health services. 140 persons (25%) have been in contact with a primary care health centre, 101 persons (18%) with out-patient specialist mental health care and 58 persons (10%) with a private practitioner. Some of them had sought help from many sources.

### Attitudes connected with depression

Logistic regression analysis showed that female gender and younger age predicted major depression (Table 2). Also, less desire for social distance and positive attitudes towards antidepressants predicted the occurrence of depression. The "Depression is a matter of will"- scale did not have a statistically significant connection with depression. In this model the Nagelkerke *R*^2 ^was 0.07.

**Table 2:** Logistic associations between gender, age, attitude scales and depression (n = 4401)

	Odds ratio (95% CI)
Gender (female)	1.82 (1.47-2.24) ***
Age (year of birth)	1.02 (1.01-1.02) ***
"Depression is a matter of will" scale ^1^	1.03 (0.99-1.07)
"Desire for social distance" scale^1^	0.82 (0.77-0.87) ***
"Antidepressant attitudes" scale^1^	0.91 (0.85-0.98)*

Nagelkerke *R*^2^	0.07

*P < 0.05; **P < 0.01; ***P < 0.001

^1 ^Scale is standardized by the mean and std. deviation of the whole sample

### Attitudes connected with use of mental health services among people with depression

In the logistic regression analysis where the use of mental health services was the dependent variable female gender, higher age and more serious degree of depression predicted more active service use among those with depression (Table 3). Less desire for social distance predicted more active service use as well as positive attitudes towards antidepressants. In this model the Nagelkerke *R*^2 ^was 0.21.

**Table 3:** Logistic associations between gender, age, attitude scales and mental health service use among people with depression (n = 507)

	Odds ratio (95%CI)
Gender (female)	1.65 (1.06-1.82)*
Age (year of birth)	0.98 (0.97-1.00) *
Depression severity	1.24 (1.06-1.47) **
"Depression is a matter of will" scale ^1^	0.95 (0.89-1.03)
"Desire for social distance" scale^1^	0.81 (0.73-0.90) ***
"Antidepressant attitudes" scale^1^	0.62 (0.54-0.72) ***

Nagelkerke *R*^*2*^	0.21

*P < 0.05; **P < 0.01; ***P < 0.001

^1 ^Scale is standardized by the mean and std. deviation of the whole sample

## Discussion

To our knowledge this is the first large population study in Europe that investigates the connection between stigmatizing attitudes and actual use of mental health services among those with depression.

Some limitations of our study need to be considered. First, the survey response rate was 51.6%. It is however increasingly difficult to reach higher response rates in mail surveys of the general population, and it has been claimed that percentages over 50 are acceptable and even in some cases good [28]. In our data the risk of non-response bias is highest among the young, with the response rate was below 40% for those aged 16-23 and also among men, whose overall response rate was 43%. Second, because we chose to customize the attitude and discrimination scales for our population we must be careful when comparing our results with earlier studies. However, many individual scale items were identical with items used in previous stigma studies. The internal consistency of our depression stigma- and discrimination-scales is acceptable if we take into the consideration the shortness of our scales [29]. Third, in some attitude items we use such vague expressions as "mental health problem" or "mental illness" which can be perceived in different ways by respondents. It is possible that a person with depression does not think that he or she has a "mental health problem". We also know that stereotypes connected with different mental conditions can vary a lot [30]. Fourth, this study is a cross-sectional study and cannot be taken as providing evidence of causal relationship between the attitude items and scales and professional help-seeking. People's experiences of health care services probably have an effect on their attitudes as has been shown in previous studies [31,32]. Finally, social desirability may always have an effect on attitude questionnaires. People are likely to underreport their stigmatizing stereotypes compared with their real-life behavior. In our social distance scale we measure people's intentions, not their actual behavior.

When inspecting the actual self-reported professional service use among those with depression, more active use of services is connected with realistic views on the effects of antidepressants and fewer discriminative social intentions. Interaction between the severity of depression and stigma may also have an important role in mental health service use.

Occurrence of depression and personal beliefs about one's own responsibility for depression did not correlate. One might expect people with depression to be aware that they are not responsible for their problems, but our results suggest that many of them also share the stereotypes prevailing in society and maybe stigmatize themselves. An alternative explanation for this result is depression itself. Self-accusation is one of the typical symptoms in depression and it may counteract the personal knowledge about the nature of origins of depression.

On the social discrimination scale, people with depression showed more social tolerance towards people with mental problems. This replicates results from previous studies [33,34]. The greater the knowledge of or experience with mental illness, the less frequently people express the desire to keep social distance from people with mental conditions. Perhaps experiencing the burden of depression helps one empathize with the suffering of other people.

Those with depression seem to know more about the non-addictive nature of antidepressants, possibly because of their own experiences of those medicines.

Almost 40% of persons with questionnaire scores indicating major depressive disorder had had contact with health care professionals during the last year. Internationally this is a rather positive result but far from optimal. Another result was also alarming: the prevalence of depression was higher among younger people, but older people used services more actively.

In our data, respondents with more serious depression had used mental health services more actively. This connection has been found in previous studies too [35,36].

It can be assumed that if a person believes that he is responsible for his depression, he bears more feelings of guilt and shame and hesitates to seek professional help. In our data this hypothesis was not confirmed. "Depression is a matter of will" - scale was not connected to service use.

If respondents with depression say they are willing to have close social contact with people with mental problems, their probability of using mental health services was higher. This connection has been found at least in one earlier study [17]. Perhaps people with depression are not worried about the perceived public stigma associated with seeking professional services if they have had contact with someone who has experienced mental problems. Attitudes toward antidepressant drugs seem to be an important differentiating factor between those who use mental health services for their depression and those who do not. Knowledge or belief about the adverse effects of antidepressants is relevant but even more so is the worry about addiction. This worry may connect with the idea of "self management" and that many people are afraid of all kinds of dependence - also in therapeutic relationships. On a primary health care level, the role of attitudes towards antidepressants is especially important because psychotherapy is often unavailable.

## Conclusions

Although stronger discriminative intentions can reduce the use of mental health services our data suggests that this does not necessarily prevent professional service use if depression is serious and views about antidepressant medication are realistic.

One important target in public health campaigns should be to improve people's knowledge about anti-depressant medication. The beliefs about plentiful side effects and a high risk of becoming addicted to antidepressants needs clarification in people's minds, because those ideas may have a connection with professional help seeking. The impact of addressing these topics in public campaigns should be evaluated in future research.

## Competing interests

The authors declare that they have no competing interests.

## Authors' contributions

All authors have read and approved the final manuscript. EA conceived the study, performed the statistical analysis and drafted the manuscript. AT revised the statistical analysis. JT and KW were involved in critically revising the manuscript for important intellectual content and data acquisition.

## Pre-publication history

The pre-publication history for this paper can be accessed here:

http://www.biomedcentral.com/1471-244X/11/52/prepub
